# Myeloid Sarcoma Developing in Prexisting Hydroxyurea-Induced Leg Ulcer in a Polycythemia Vera Patient

**DOI:** 10.1155/2013/497593

**Published:** 2013-09-16

**Authors:** Hatim Nafil, Illias Tazi, Lahoucine Mahmal

**Affiliations:** Service d'Hématologie, CHU Mohamed VI, Université Cadi Ayyad Marrakech, Marrakech 40000, Morocco

## Abstract

Myeloid sarcoma (MS) is an extramedullary tumour consisting of myeloblasts or immature myeloid cells located in an extramedullary site. It may occur at presentation of AML, at relapse, or prior to the onset of frank leukemia. We report a rare case of MS developing in prexisting Hydroxyurea-induced leg Ulcer in a 70-year-old woman.

A 78-year-old Moroccan woman with polycythemia vera on hydroxyurea therapy 1 g/day, for 5 years duration was admitted to our department because of painful ulcer over the right lateral malleolus. The physical examination showed ulceration of the malleous measuring 2 × 3 cm with no evidence of peripheral neuropathy. The lower limb pulsations were well felt and there were no varicose veins. The Doppler study showed normal study of deep venous system of lower limbs with no evidence of saphenofemoral or saphenopopliteal incompetence. At this time, complete blood cell count was normal. Bacterial and fungal cultures revealed no growth. The diagnosis of Hydroxyurea-induced leg ulcer was suspected. After one month of the cessation of hydroxyurea and local treatment (local antibiotics, compression stockings, and daily wound dressing), no improvement has been objectified. A biopsy of the ulcer with a histological study was performed and showed granulocytic sarcoma with myeloblasts CD117, CD45, vimentin, and myeloperoxidase positivity, but it showed CD34, CD61, CD235a, CD30, CD7, and CD56 negativity. Laboratory investigations showed a haemoglobin level of 9 g/dL (normal range 12–16 g/dL), a platelet count of 80,000/L (150,000–400,000/L), and a white blood count (WBC) of 3,500/L (4,000–10,000/L) with 69% neutrophils, 22% lymphocytes, 7% monocytes, 1% eosinophils, and 1% basophils. Bone marrow aspiration showed transformation into AML with 46% myeloblasts with a normal karyotype. Given the patient's frailty, the patient received palliative treatment with platelet and red cell concentrates. Chemotherapy could not be performed because of poor general condition of the patient. Her general condition deteriorated and the patient died 2 months after diagnosis. We describe here a patient with Hydroxyurea-induced leg ulcer transforming to Myeloid sarcoma (MS) at the time of blastic transformation of polycythemia vera (PV) into acute myeloid leukaemia (AML).

Hydroxyurea is a cytostatic agent commonly used in the treatment of chronic myeloproliferative diseases, such as chronic myeloid leukemia, polycythemia vera, and essential thrombocytosis. Long-term hydroxyurea therapy has been associated with cutaneous side effects such as alopecia, diffuse hyperpigmentation, skin atrophy, nail changes, and erythema [1]. Leg ulceration following hydroxyurea is less common. Hydroxyurea treatment induces ulcerative skin lesion formation secondary to direct cytologic damage. Because hydroxyurea kills proliferating cells during the synthesis phase of the cell cycle, keratinocyte and collagen fiber syntheses could be impaired [2]. Leg ulcers have been noted in association with long-term hydroxyurea administration. Nine percent of patients taking hydroxyurea medication develop this complication [3]. Furthermore, the lesions disappear after the hydroxyurea is discontinued [4].

Myeloid sarcoma (MS) is an extramedullary tumour consisting of myeloblasts or immature myeloid cells located in an extramedullary site. It may occur at presentation of AML, at relapse, or prior to the onset of frank leukemia. Clinically, it presents either as single or multiple tumours in lymphoid organs, bone, skin, mucosa, and other organs [5]. 

To our knowledge, an MS developing in an already existing polycythemia vera as the first sign of an AML has not been described before. Our case underlines the importance of biopsy in the case of nonhealing ulcers, especially in patients with underlying haematological disease.
